# The cis-Regulatory Element of SNCA Intron 4 Modulates Susceptibility to Parkinson’s Disease in Han Chinese

**DOI:** 10.3389/fgene.2020.590365

**Published:** 2020-10-23

**Authors:** Shi-Guo Zhu, Hui Lu, Miao Mao, Zhao-Feng Li, Lei Cui, Begench Ovlyakulov, Xiong Zhang, Jian-Hong Zhu

**Affiliations:** ^1^Department of Geriatrics and Neurology, The Second Affiliated Hospital and Yuying Children’s Hospital, Wenzhou Medical University, Wenzhou, China; ^2^Department of Preventive Medicine, Wenzhou Medical University, Wenzhou, China

**Keywords:** Parkinson’s disease, SNCA, cis-regulatory element, association, variant

## Abstract

**Objective:** A novel functional cis-regulatory element (CRE) located at *SNCA* intron 4 has recently been identified in association with Parkinson’s disease (PD) risk in European descendants. We aimed to investigate whether this CRE is associated with PD in Han Chinese ethnicity.

**Methods:** A Chinese cohort comprising 513 sporadic PD patients and 517 controls was recruited. CRE variants were identified by sequencing and then analyzed.

**Results:** A total of nine variants were detected, namely eight single nucleotide variants and one new insertion variant. Two variants, rs17016188 and rs7684892, had minor allele frequency greater than 5%. A difference of rs17016188 was observed in males with the C allele serving as a recessive risk factor (*p* = 0.001, OR = 2.349, 95% CI = 1.414–3.901) following Bonferroni correction. Haplotypes of rs17016188 and rs7684892 showed distribution differences in the total and the male populations (*p* = 0.002 and 4.08 × 10^−5^, respectively). Among the haplotypes, rs17016188/T-rs7684892/G was associated with a reduced risk for PD (*p* = 4.8 × 10^−4^, OR = 0.731, 95% CI = 0.614–0.872).

**Conclusions:** Our results provide insight into how the *SNCA* intron 4 CRE harbors variants and its contribution to PD risk in Chinese ethnicity.

## Introduction

Parkinson’s disease (PD) is a common neurodegenerative disease characterized by the progressive loss of dopaminergic neurons in the substantia nigra and the formation of Lewy bodies. Considerable progress has been made to understand the genetic underpinning of PD. Monogenic mutations such as in genes of *SNCA*, *LRRK2*, and *Parkin* have been well defined to cause hereditary PD (Obeso et al., 2017). A number of independent risk variants including *SNCA*/rs356182, *RIT2*/rs12456492, and *SIPA1L2*/rs10797576 have been consistently identified to associate with sporadic PD (Nalls et al., 2014; Wang et al., 2015; Zou et al., 2018).

*SNCA* and its product α-synuclein are at the convergence of PD genetically and pathologically. It has been intensively investigated in regard with its causative genetic mutations and risk variants. Patients with *SNCA* duplication or triplication exhibit a dosage effect in disease onset, severity, and progression (Obeso et al., 2017). The encoded α-synuclein is the main component of Lewy body inclusion, and dopaminergic neurons are particularly vulnerable to the toxic α-synuclein aggregates (Alegre-Abarrategui et al., 2019). In recent years, misfolded α-synuclein is considered possessing prion-like properties, propagating between neurons, neurons and glia cells, and even between animals (Makin, 2016).

By analyzing open chromatins of midbrain dopaminergic neurons and their correlations with putative enhancers, McClymont et al. (2018) recently revealed a novel functional cis-regulatory element (CRE; chr4: 90,721,063–90,722,122; hg19) located at *SNCA* intron 4. Variants of this CRE were shown to be associated with elevated PD risk in non-Hispanic Caucasians of European descent and be able to impact DNA-binding capacity of a set of proteins. Also, DNase hypersensitivity site analysis showed an interaction between the CRE and the *SNCA* promoter (Thurman et al., 2012; Wang et al., 2018). However, whether this *SNCA* CRE is associated with PD risk in other ethnicities remains unknown. In this study, we recruited a total of 1,020 individuals of Han Chinese descent and aimed to investigate those bearing CRE variants and their associations with PD risk.

## Materials and Methods

### Subjects

Enrolled in this study were 513 sporadic PD patients (264 males and 249 females) and 507 controls (287 males and 220 females) of Han Chinese ethnicity from southeast China. The median ages of the patients and controls were 64 (interquartile range, 55–71) and 65 (interquartile range, 60–73) years, respectively, following a normality test. PD patients were diagnosed by two movement disorder specialists according to the UK Parkinson’s disease Society Brain Bank Criteria. Patients with a family history of PD or with secondary and atypical Parkinsonism were excluded. Control subjects were free of neurological disorders according to medical history and physical and laboratory examinations. All subjects participating in the study signed written informed consent. The study was approved by the Ethics Committee of the Second Affiliated Hospital and Yuying Children’s Hospital, Wenzhou Medical University (No. KYKT2018-15, 03/02/2018).

### Genotyping

Genomic DNA was extracted from human peripheral blood using an extraction kit from Tiangen (Beijing, China). The CRE fragment was amplified by polymerase chain reaction (PCR) in 20 μl of reaction mixture that contained 2 μl DNA template, 10 μl 2 × PCR Master Mix (Vazyme, Nanjing, China), and 1 μl of each primer. The primer pair was 5'-GGG AGG GAA CTA TGG AGG CAT C-3' and 5'-GGC CTA GCA GGG CGA GAA TAG-3'. The PCR condition was initial denaturation at 95°C for 3 min, followed by 30 cycles of 95°C for 30 s, 60°C for 30 s, and 72°C for 60 s, and a final extension at 72°C for 5 min. Variants were identified by bidirectional Sanger sequencing (BGI, Shanghai, China).

### Data Analysis

Sequence data were analyzed using Lasergene (DNASTAR). Statistical analyses were performed using the statistical package of Predictive Analytics Software 18.0 (PASW, version 18.0) for Windows. Hardy-Weinberg equilibrium in genotype distribution, as well as differences in gender, genotype, allele, and haplotype frequencies, was assessed using the *χ*^2^ test. Mann-Whitney *U* test was used to evaluate age difference. Variants with significant difference were further analyzed using binary logistic regression model with gender and age as covariates. Haplotype association analysis was evaluated using SHEsis (Shi and He, 2005; Li et al., 2009). Linkage disequilibrium structure and *r*^2^ values of the *SNCA* loci were extracted from LDlink with the LDmatrix tool in the 1,000 Genomes Chinese population (Machiela and Chanock, 2015) and were plotted with R.^1^ Statistical significance was considered when *p* < 0.05.

## Results

### Identification of Variants in the CRE Region of SNCA Intron 4

A total of nine variants were detected in the CRE region out of the 1,020 subjects (Table 1). Among these variants, eight were single nucleotide variation and one was insertion variation. Two single nucleotide variants, rs17016188 and rs7684892, had minor allele frequencies greater than 5%. The other six single nucleotide variants were found in heterozygous carriers. Based on the TOPMed project,^2^ the chr4: 90,721,599 (G > A) discovered in a PD patient was a new variant, and the rs1245859102 discovered in a control was a new G > T transversion instead of the usual G > A transition. The homozygous insertion variant, chr4: 90,722,259, was discovered in a control subject (Table 1).

**Table 1:** Identified variants and their allele and genotype frequencies in PD cases and controls.

Variant	Minor allele	Subject	Allele, *n* (%)		Genotype, *n* (%)		
			Minor	Major	Hm minor	Heterozygous	Hm major
chr4: 90,722,259 (insertion)	GAGGAA	Control	2 (0.2)	1,013 (99.8)	1 (0.2)	0 (0.0)	506 (99.8)
		PD	0 (0.0)	1,026 (100.0)	0 (0.0)	0 (0.0)	513 (100.0)
rs1245859102 (G/T)	T	Control	1 (0.1)	1,013 (99.9)	0 (0.0)	1 (0.2)	506 (99.8)
		PD	0 (0.0)	1,026 (100.0)	0 (0.0)	0 (0.0)	513 (100.0)
rs2737024 (A/G)	G	Control	0 (0.0)	1,014 (100.0)	0 (0.0)	0 (0.0)	507 (100.0)
		PD	2 (0.2)	1,024 (99.8)	0 (0.0)	2 (0.4)	511 (99.6)
chr4: 90,721,599 (G/A)	A	Control	0 (0.0)	1,014 (100.0)	0 (0.0)	0 (0.0)	507 (100.0)
		PD	1 (0.1)	1,025 (99.9)	0 (0.0)	1 (0.2)	512 (99.8)
rs2583959 (C/G)	G	Control	0 (0.0)	1,014 (100.0)	0 (0.0)	0 (0.0)	507 (100.0)
		PD	2 (0.2)	1,024 (99.8)	0 (0.0)	2 (0.4)	511 (99.6)
rs17016188 (T/C)	C	Control	345 (34.0)	669 (66.0)	61 (12.0)	223 (44.0)	223 (44.0)
		PD	392 (38.2)	634 (61.8)	86 (16.8)	220 (42.9)	207 (40.4)
rs7684892 (G/A)	A	Control	190 (18.7)	824 (81.3)	18 (3.6)	154 (30.4)	335 (66.1)
		PD	228 (22.2)	798 (77.8)	27 (5.3)	174 (33.9)	312 (60.8)
rs1389820774 (C/T)	T	Control	1 (0.1)	1,013 (99.9)	0 (0.0)	1 (0.2)	506 (99.8)
		PD	5 (0.5)	1,021 (99.5)	0 (0.0)	5 (1.0)	508 (99.0)
rs1560531460 (T/A)	A	Control	1 (0.1)	1,013 (99.9)	0 (0.0)	1 (0.2)	506 (99.8)
		PD	1 (0.1)	1,025 (99.9)	0 (0.0)	1 (0.2)	512 (99.8)

*Hm*, homozygous; *PD*, Parkinson’s disease.

### Association Analysis of the Variants rs1701688 and rs7684892 With PD Risk

Genotype distribution of both rs17016188 and rs7684892 met with Hardy-Weinberg equilibrium (*p* > 0.05). The gender frequencies in the PD cases and controls were comparable (*p* > 0.05). There was a difference in age between the two groups (*p* < 0.05). Allele frequencies of both rs17016188 and rs7684892 showed marginal associations with PD risk in the total population (*p* = 0.049 and 0.051, respectively; Table 2). After Bonferroni correction, the difference, that is *p* < 0.025, was found in the male group in rs17016188 (allele: *p* = 0.004, OR = 1.433, 95% CI = 1.120–1.833; genotype: *p* = 0.003). Further multivariate analysis of rs17016188 with age adjusted confirmed the difference in the males with the C allele serving as a recessive risk factor (*p* = 0.001, OR = 2.349, 95% CI = 1.414–3.901; Table 3).

**Table 2:** Association analysis of rs17016188 and rs7684892 with PD risk.

Variant	Allele, *n* (%)		OR (95% CI)	*p*	Genotype, *n* (%)			*p*
rs17016188	T	C			TT	CT	CC	
Control	669 (66.0)	345 (34.0)			223 (44.0)	223 (44.0)	61 (12.0)	
PD	634 (61.8)	392 (38.2)	1.199 (1.001–1.437)	0.049	207 (40.4)	220 (42.9)	86 (16.8)	0.089
Male control	388 (67.6)	186 (32.4)			127 (44.3)	134 (46.7)	26 (9.1)	
Male PD	313 (59.3)	215 (40.7)	1.433 (1.120–1.833)	0.004^*^	99 (37.5)	115 (43.6)	50 (18.9)	0.003^*^
Female control	281 (63.9)	159 (36.1)			96 (43.6)	89 (40.5)	35 (15.9)	
Female PD	321 (64.5)	177 (35.5)	0.974 (0.746–1.273)	0.850	108 (43.4)	105 (42.2)	36 (14.5)	0.884
rs7684892	G	A			GG	AG	AA	
Control	824 (81.3)	190 (18.7)			335 (66.1)	154 (30.4)	18 (3.6)	
PD	798 (77.8)	228 (22.2)	1.239 (0.999–1.537)	0.051	312 (60.8)	174 (33.9)	27 (5.3)	0.149
Male control	474 (82.6)	100 (17.4)			196 (68.3)	82 (28.6)	9 (3.1)	
Male PD	409 (77.5)	119 (22.5)	1.379 (1.025–1.856)	0.033	163 (61.7)	83 (31.4)	18 (6.8)	0.079
Female control	350 (79.6)	90 (20.5)			139 (63.2)	72 (32.7)	9 (4.1)	
Female PD	389 (78.1)	109 (21.9)	1.090 (0.796–1.492)	0.592	149 (59.8)	91 (36.8)	9 (3.6)	0.680

*CI*, confidence interval; *OR*, odds ratio; *PD*, Parkinson’s disease.

* Indicates *p* < 0.025 after Bonferroni correction.

**Table 3:** Multivariate analysis of rs17016188 in the male PD cases and controls adjusted with age.

rs17016188	Genotype, *n* (%)			OR (95% CI)	*p*
	TT	CT	CC		
Control	127 (44.3)	134 (46.7)	26 (9.1)		
PD	99 (37.5)	115 (43.6)	50 (18.9)		
Additive				1.425 (1.114–1.823)	0.005^*^
Dominant				1.323 (0.941–1.861)	0.108
Recessive				2.349 (1.414–3.901)	0.001^*^

*CI*, confidence interval; *OR*, odds ratio; *PD*, Parkinson’s disease.

* Indicates *p* < 0.05.

### Haplotype Analysis of rs1701688 and rs7684892 in Association With PD

Haplotypes were constructed to understand the combined effect of rs17016188 and rs7684892 on PD risk (Table 4). The two variants displayed a linkage disequilibrium with *r*^2^ = 0.146 and *D’* = 0.9993 in the cohort. Out of four potential combinations, three haplotypes were detected except for the rs17016188/C-rs7684892/A. An overall significance in haplotype distribution was found in the total and male populations (*p* = 0.002 and 4.08 × 10^−5^, respectively), but not in the females. The haplotype, rs17016188/T-rs7684892/G, exhibited a protective effect against PD in the total population (*p* = 4.8 × 10^−4^, OR = 0.731, 95% CI = 0.614–0.872) and in the males (*p* = 7.14 × 10^−6^, OR = 0.577, 95% CI = 0.453–0.734).

**Table 4:** Haplotype frequencies of rs17016188 and rs7684892 in PD cases and controls.

rs17016188	rs7684892	Control, *n* (%)	PD, *n* (%)	OR (95% CI)	*p*
Total					
T	G	479 (47.2)	406 (39.6)	0.731 (0.614–0.872)	4.8 × 10^–4^^*^
C	G	345 (34.0)	392 (38.2)	1.199 (1.001–1.437)	0.049
T	A	190 (18.7)	228 (22.2)	1.239 (0.999–1.538)	0.051
Total		1,014 (100)	1,026 (100)		0.002^*^
Men					
T	G	288 (50.2)	194 (36.7)	0.577 (0.453–0.734)	7.14 × 10^–6^^*^
C	G	186 (32.4)	215 (40.7)	1.433 (1.120–1.833)	0.004^*^
T	A	100 (17.4)	119 (22.5)	1.380 (1.025–1.857)	0.033
Total		574 (100)	528 (100)		4.08 × 10^–5^^*^
Women					
T	G	191 (43.4)	212 (42.6)	0.966 (0.746–1.252)	0.796
C	G	159 (36.1)	177 (35.5)	0.974 (0.746–1.273)	0.850
T	A	90 (20.5)	109 (21.9)	1.090 (0.796–1.492)	0.592
Total		440 (100)	498 (100)		0.866

*CI*, confidence interval; *OR*, odds ratio; *PD*, Parkinson’s disease.

* Indicates *p* < 0.017 after Bonferroni correction.

**Figure 1:**
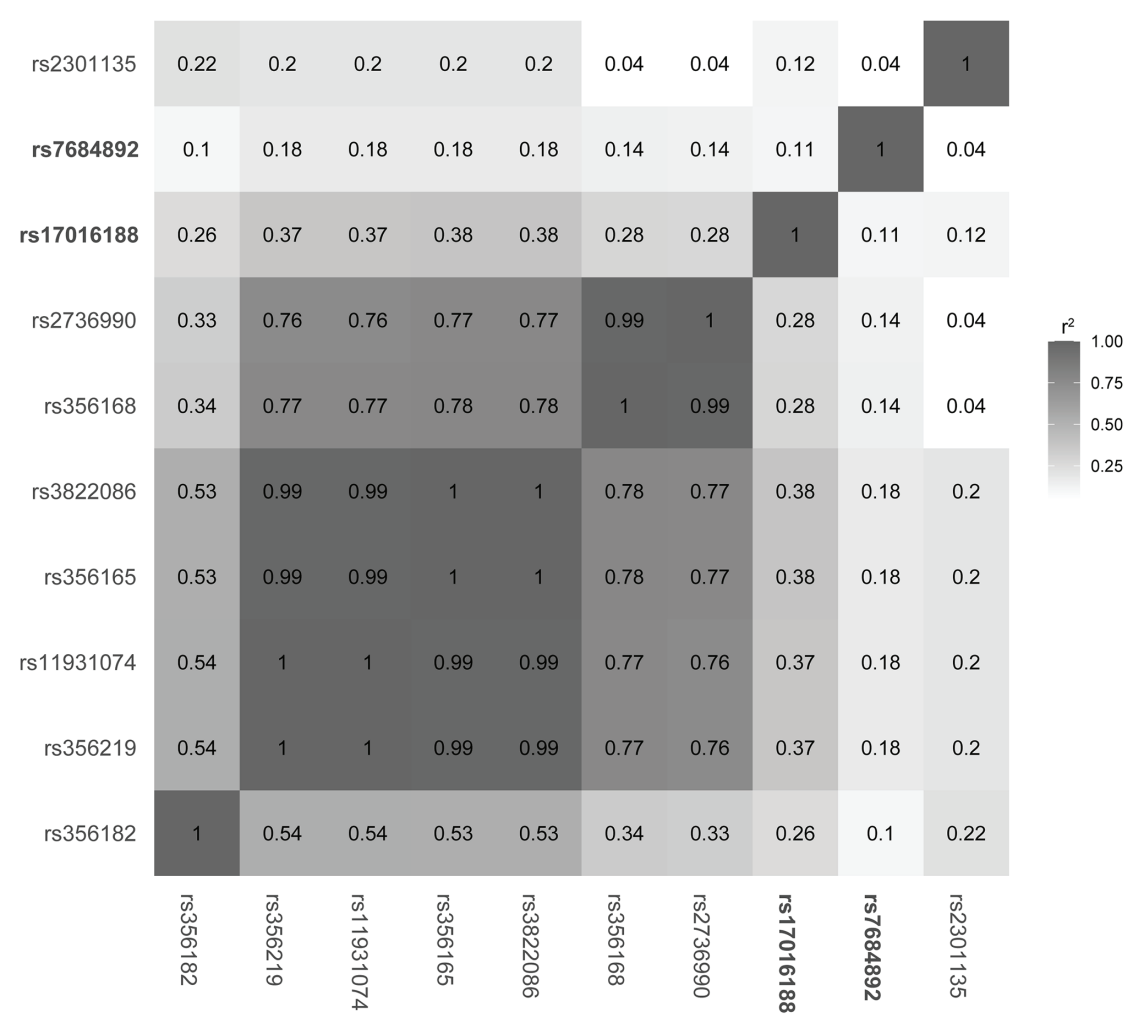
Linkage disequilibrium structure of the *SNCA* loci.

Linkage disequilibrium structure was subsequently constructed in the 1,000 Genomes data to understand how the two CRE variants might be interacting with other *SNCA* variants (Figure 1). Of the variants, rs356168 is a previous functionally validated variant (Soldner et al., 2016) and rs356182 is the lead variant of genome-wide associated studies (GWAS; Nalls et al., 2014; Chang et al., 2017). The rest are a panel of PD-associated variants identified across *SNCA* in Chinese ethnicity (Li et al., 2019). The linkage disequilibrium analysis showed a low linkage disequilibrium structure between the CRE variants (rs1701688 and rs7684892) and the previously reported *SNCA* sites.

## Discussion

**Figure 2:**
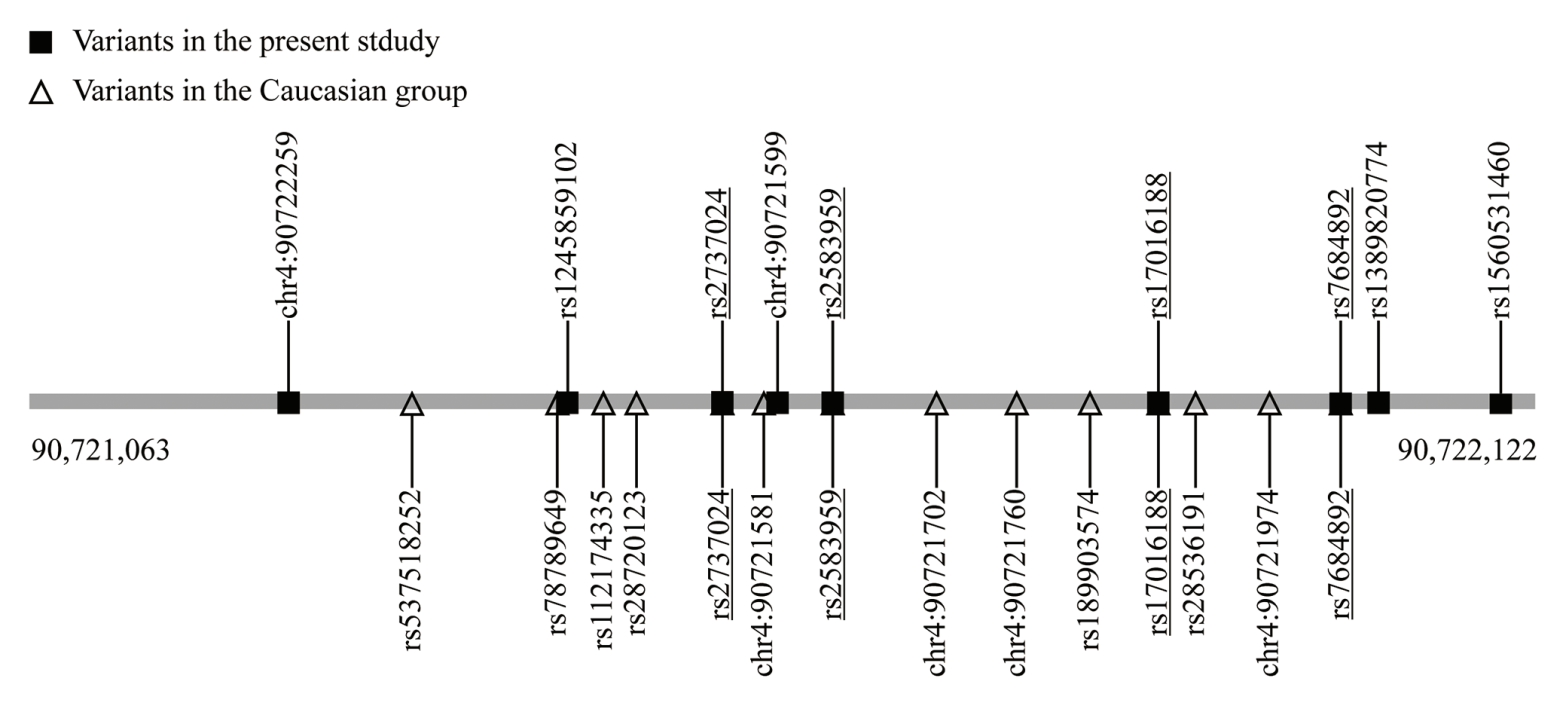
The variants in the *SNCA* intron 4 CRE. The underlined are the shared variants.

*SNCA* is a central piece of the genetic puzzle leading to PD pathogenesis. The recent advance of the *SNCA* intron 4 CRE in association with PD risk warrants further identification in additional ethnicities. The current study involving a Chinese population reveals nine variants, including one new rare variant (chr4: 90,721,599; G > A) and one variant with different nucleotide replacement (rs124589102; G > T). Four variants, namely rs17016188, rs7684892, rs2737024, and rs2583959, are common between ours and the 14 variants discovered in the European descendants (McClymont et al., 2018; Figure 2).

Current knowledge of biologically related noncoding variation is limited. It is considered that CRE candidates may serve functional roles in regulating gene expression (Snyder et al., 2020). Recent studies have highlighted the importance of CREs harboring functional variation as well as their regulated genes (Maurano et al., 2012; Thurman et al., 2012; Soldner et al., 2016; McClymont et al., 2018). For instance, the CRE in *SNCA* intron 4 may affect the midbrain-specific expression of *SNCA*, and the variants within the CRE can disturb binding affinity of a set of proteins, including NOVA1, APOBEC3C, PEG10, SNRPA, and CHMP5. Three of them, PEG10, SNRPA, and CHMP5, show increased affinities of binding to the minor risk allele, that is, G of rs2583959 or G of rs2737024. To understand whether the CRE variants are independently connected to PD, eight known PD-associated *SNCA* risk variants, including those functionally validated and leading in GWAS studies (Nalls et al., 2014; Soldner et al., 2016; Chang et al., 2017), and those identified in a Chinese population (Li et al., 2019), are used for linkage disequilibrium analysis. Results suggest that the CRE variants rs1701688 and rs7684892 and the resulting haplotypes are independent in association with PD. Though detailed mechanisms and consequences remain to be further explored, it is possible that the variants within the CRE, independently or in concert with additional variation, may influence *SNCA* expression and/or susceptibility of catecholaminergic neurons.

In line with the previous results (McClymont et al., 2018), both rs17016188 and rs7684892 show minor allele frequency over 5% and are not associated with PD risk in the overall population. While the gender-stratified analysis was not presented in the earlier study, our results suggest an impact of rs17016188 on PD risk in males. Similar results were also found in the PD-associated protective haplotype, rs17016188/T-rs7684892/G. It is not clear how gender bias occurs in the *SNCA* CRE variant and haplotype, which may need further verification in other populations. Nonetheless, the gender bias per se is not surprising for genetic association with PD risk (Palacios et al., 2010; Klebe et al., 2013; Wang et al., 2015). The male-to-female ratio of PD prevalence is approximately at 3:2 (de Lau and Breteler, 2006), probably due to genes in the sex chromosomes and sex hormone-related mechanisms (Jurado-Coronel et al., 2018).

The other two variants, rs2737024 and rs2583959, whose minor allele frequencies are above 5% in the Caucasian population, are significantly associated with PD risk (Heckman et al., 2012; McClymont et al., 2018). In contrast, it is reported that rs2583959 is not associated with multiple system atrophy and essential tremor (Al-Chalabi et al., 2009; Ross et al., 2011). Interestingly, both variants are rare single nucleotide polymorphisms in the Chinese population. Each variant is only found in two PD patients in heterozygous form, and coincidentally, their carriers are identical individuals. Although their frequencies are distinct between the Chinese and Caucasian populations, our results support the previous findings that the minor alleles of rs2737024 and rs2583959 aggravated PD risk and the two variants were in strong linkage disequilibrium (McClymont et al., 2018).

In conclusion, the current study reveals nine variants in the *SNCA* intron 4 CRE and demonstrates their associations with PD risk in a Chinese population. Our results provide further insight into how this specific gene regulatory region of *SNCA* harbors variants contributing to PD risk in different ethnicities.

## Data Availability Statement

The original contributions presented in the study are included in the article/supplementary material, further inquiries can be directed to the corresponding authors.

## Ethics Statement

The studies involving human participants were reviewed and approved by the Ethics Committee of the Second Affiliated Hospital and Yuying Children’s Hospital, Wenzhou Medical University. The patients/participants provided their written informed consent to participate in this study.

## Author Contributions

J-HZ and XZ designed the research. XZ, S-GZ, and BO collected and managed the samples. S-GZ, HL, and MM conducted the experiments. S-GZ, Z-FL, and LC analyzed the data. J-HZ and S-GZ wrote the manuscript. All authors contributed to the article and approved the submitted version.

### Conflict of Interest

The authors declare that the research was conducted in the absence of any commercial or financial relationships that could be construed as a potential conflict of interest.

## Footnotes

^1^https://www.r-project.org/

^2^https://www.nhlbiwgs.org
